# Application of Small Molecules in the Central Nervous System Direct Neuronal Reprogramming

**DOI:** 10.3389/fbioe.2022.799152

**Published:** 2022-07-07

**Authors:** Jingyi Wang, Shiling Chen, Chao Pan, Gaigai Li, Zhouping Tang

**Affiliations:** Department of Neurology, Tongji Hospital, Tongji Medical College, Huazhong University of Science and Technology, Wuhan, China

**Keywords:** small molecule, direct neuronal reprogramming, transdifferentiation, regeneration medicine, neuron, neurological disease

## Abstract

The lack of regenerative capacity of neurons leads to poor prognoses for some neurological disorders. The use of small molecules to directly reprogram somatic cells into neurons provides a new therapeutic strategy for neurological diseases. In this review, the mechanisms of action of different small molecules, the approaches to screening small molecule cocktails, and the methods employed to detect their reprogramming efficiency are discussed, and the studies, focusing on neuronal reprogramming using small molecules in neurological disease models, are collected. Future research efforts are needed to investigate the *in vivo* mechanisms of small molecule-mediated neuronal reprogramming under pathophysiological states, optimize screening cocktails and dosing regimens, and identify safe and effective delivery routes to promote neural regeneration in different neurological diseases.

## 1 Introduction

The regenerative repair of neurons in acute injuries and neurodegeneration diseases has been clinically challenging (Sivandzade and Cucullo, 2021). The main reason for this is the extremely limited neuron-regenerative capacity in the adult mammalian Central Nervous System (CNS), which contributes to the poor prognosis of patients with CNS injuries (Goldman, 2016; Tedeschi et al., 2016; McMurran et al., 2018; Vignoles et al., 2019; Stricker and Gotz, 2021). In the last decades, stem cell transplantation therapy has been proposed to be an efficient method of replacing lost neurons in several neurological disorders (Albert et al., 2021; Lu et al., 2021; Salikhova et al., 2021). However, some existing problems, such as teratogenic effect, differentiation abnormality, ethical issues, and survival limitation, prevent its clinical appilications (Barker and de Beaufort, 2013; Qin et al., 2017; Zeng et al., 2021). Recently, an emerging reprogramming technique that transforms non-neuronal cells into induced neurons (iNs) can be regarded as a promising potential therapeutic tool in regenerative medicine (Ma et al., 2019). Instead of exogenous cells’ transplantation, this technique makes it possible to directly reprogram endogenous starting cells into target cells *in vivo* (Srivastava and DeWitt, 2016).

**FIGURE 1:**
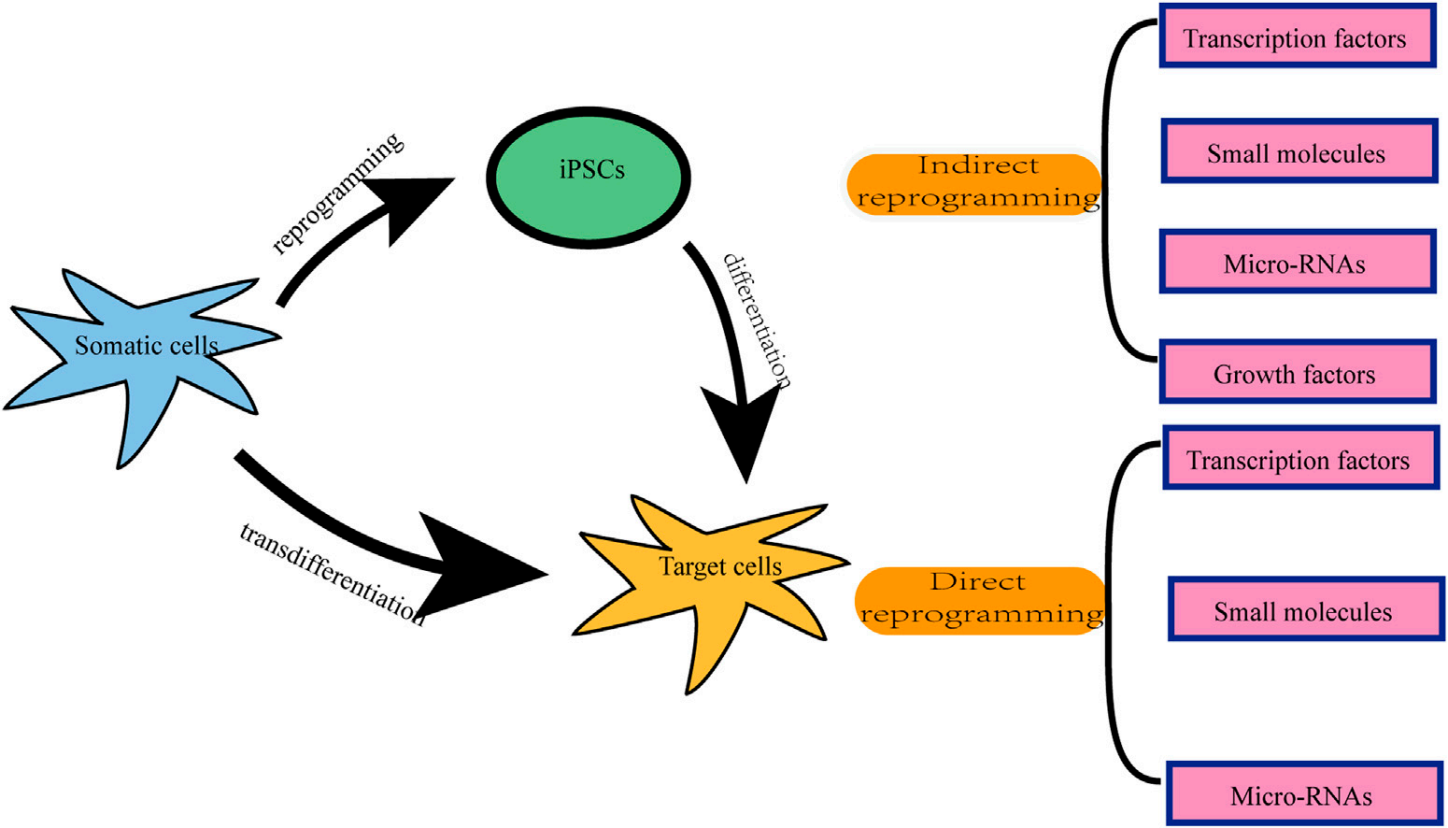
Schematic diagram of two types of reprogramming. iPSCs: induced pluripotent stem cells.

There are two types of neuronal reprogramming: indirect reprogramming and direct reprogramming (Figure 1) (Sato et al., 2019). The former is a two-step process, in which differentiated somatic cells are first reprogrammed into intermediated states such as induced pluripotent stem cells (iPSCs) or multipotent neural stem cells (iNSCs), followed by differentiation into neurons (Kim et al., 2011; Ladewig et al., 2013; Liu et al., 2016; Yuan et al., 2020). The latter is the direct transformation of terminally differentiated cells into target mature cells without passing through the stages of pluripotent or multipotent cells, which is also known as transdifferentiation (Kim et al., 2011; Ladewig et al., 2013; Liu et al., 2016; Mollinari et al., 2018; Yang et al., 2020a; Yuan et al., 2020; Wang et al., 2021a). Since direct lineage reprogramming does not go through an intermediate stem cell state, it possesses the appealing features of a low likelihood of tumor formation, no age-resetting, a high conversion speed, and an efficient cell differentiation (Yang et al., 2011; Li and Chen, 2016; Ma et al., 2019; Mollinari and Merlo, 2021).

There are mainly several approaches to achieving reprogramming, each with its advantages and disadvantages. One involves reprogramming mediated by the ectopic gene expression, such as the ectopic expression of neuronal transcription factors (TFs) *via* virus vectors (Vierbuchen et al., 2010; Meng et al., 2012). As for endogenous gene manipulation, an emerging and innovative technology, clustered regularly interspaced short palindromic repeat (CRISPR)/Cas9 system can easily edit and modulate deoxyribonucleic acid (DNA) sequences within the endogenous genome (Hsu et al., 2014). It was reported that the application of CRISPR/Cas9 can directly convert fibroblasts into neuronal cells via silencing endogenous non-neuronal genes (Rubio et al., 2016). Another different method is associated with the use of small-molecule compounds during the process of reprogramming (Dai et al., 2015; Li et al., 2015). Besides, microRNAs are included in cellular conversion formulations to aid the inhibition of alternative cell fates (Ambasudhan et al., 2011; Yoo et al., 2011; Xue et al., 2013; Zhou et al., 2015). For the last decade, as one of the small non-coding RNAs, microRNAs were combined with TFs and small molecules to kickstart the successful conversion of iNs (Ambasudhan et al., 2011; Smith et al., 2016; Habekost et al., 2020; Nemoto et al., 2020), which suppress gene expression by promoting mRNA degradation or by blocking translation (Bushati and Cohen, 2007; Krol et al., 2010). Furthermore, studies have shown that growth factors promote the process of reprogramming (He et al., 2017; Yi et al., 2021).

Reviewing the published research, the two most widely used methods in the field of reprogramming are the ectopic expression of TFs and the administration of small molecule cocktails. The overexpression of TFs using viral vectors has been reported to be efficient in mediating reprogramming (Yang et al., 2020b; Puls et al., 2020). The lineage-specific viral vectors are targeted (Brulet et al., 2017; Liang et al., 2018). The virus vector genomes can insert target cell genomes (lentiviruses and retroviruses) or form stable episomes (adeno-associated viruses) during the reprogramming, which cause TFs to sustain expression with long-term induction effects (Duan et al., 1998; Grande et al., 2013; Brulet et al., 2017; Chan et al., 2017; Morabito et al., 2017; Liang et al., 2018; Man et al., 2018; Haridhasapavalan et al., 2019). However, when integrating vectors, e.g., lentiviruses and retroviruses are used to deliver the TFs (Naldini et al., 1996; Lesbats et al., 2016), this approach may also carry the risk of causing mutations in the host cell genome (Li et al., 2002; Modlich et al., 2006; Medvedev et al., 2010); thus, raising safety issues, such as tumor formation (Oh et al., 2017; Pu et al., 2019). While the non-integrating vectors, such as adeno-associated viruses, are involved, the efficiency of reprogramming is low and the delivery capacity of TFs is limited (Shao and Wu, 2010; Hirsch et al., 2016). The other universally used approach is to induce somatic cells’ transdifferentiation using small molecules (Cheng et al., 2015a; Hu et al., 2015; Zhang et al., 2016a; Cao et al., 2016; Zeng et al., 2021). We summarized the most common small molecules during the transdifferentiation of somatic cells into neurons (Table 1). Since no exogenous genes are introduced and there is no risk of genetic manipulation, this approach seems to be a safer way of reprogramming (Cheng et al., 2015b; Hu et al., 2015; Zhang et al., 2016a; Cao et al., 2016; Zeng et al., 2021). In addition, compared with the viral vector-mediated reprogramming method, small molecules therapy has the advantages of being cost-effective, easy to obtain, reversible and controllable, strongly permeable, and lacking immunogenicity issues (Xu et al., 2008; Hou et al., 2013; Xu et al., 2015; Cao et al., 2016; Ye et al., 2016; Qin et al., 2017; Xie et al., 2017). Therefore, small molecules-mediated reprogramming has a great potential for clinical translation and is a promising therapeutic strategy for the treatment of neurological diseases. However, compared with TF-induced reprogramming, which is only required for ectopic expression of fate-determining TFs of related cells, in most cases, small molecules mediating cell reprogramming are not as specific as this latter methods (Yang et al., 2019). In general, single small molecules cannot induce somatic reprogramming, while TFs can (Yin et al., 2019). It has been demonstrated that small molecule compounds can directly convert terminally differentiated cells into neurons (Hou et al., 2013; Cheng et al., 2015b; Li et al., 2015; Zhang et al., 2015; Gao et al., 2017). Using a mixture of small molecules, somatic cells such as fibroblasts (Hu et al., 2015; Li et al., 2015; Qin et al., 2018; Wan et al., 2018; Yang et al., 2019; Xu et al., 2020), astrocytes (Zhang et al., 2015; Gao et al., 2017; Ma et al., 2019), human urine cells (Xu et al., 2019; Liu et al., 2020), peripheral blood T cells (Tanabe et al., 2018), and even glioma cells can be directly induced into neurons (Oh et al., 2017; Lee et al., 2018). In this review, we will mainly discuss small molecules-mediated direct reprogramming of the CNS neurons.

**TABLE 1:** The most common small molecules during the transdifferentiation of somatic cells into neurons.

Name of the chemicals	Main function(s)	References
A83-01	TGF-β (ALK4/5/7) signaling pathway inhibitor	Qin et al. (2018); Yang et al. (2019)
CHIR99021	GSK3 inhibitor, Wnt signaling pathway enhancer	Vierbuchen et al. (2010); Hu et al. (2015); Li et al. (2015); Zhang et al. (2015); Gao et al. (2017); Wan et al. (2018); Xu et al. (2019)
DAPT	Gamma-secretase inhibitor	Zhang et al. (2015); Yang et al. (2019); Yin et al. (2019)
DBcAMP	PKA activator	Ma et al. (2021)
Dorsomorphin	AMPK and BMP I receptor inhibitor	Hu et al. (2015); Yang et al. (2019)
DMH1	BMP inhibitor	Wan et al. (2018)
Forskolin	Adenylyl cyclase activator, cAMP/PKA signaling pathway activator, reducing lipid peroxidation	Hu et al. (2015); Li et al. (2015); Gao et al. (2017); Wan et al. (2018); Xu et al. (2019); Yang et al. (2019)
GO6983	PKC inhibitor	Hu et al. (2015); Yang et al. (2020c)
I-BET151	BET inhibitor, epigenetic reader inhibitor	Li et al. (2015); Gao et al. (2017); Lee et al. (2018); Yang et al. (2020c)
ISX−9	Neurogenesis inducer	Li et al. (2015); Gao et al. (2017); Lee et al. (2018); Yang et al. (2019); Yang et al. (2020c)
Kenpaullone	GSK3 inhibitor	Qin et al. (2018); Zhao et al. (2020)
KC7F2	HIF−1α inhibitor	Herdy et al. (2019)
LDN193189	BMP type I receptor (ALK2/3) inhibitor	Zhang et al. (2015); Hu et al. (2019); Yang et al. (2019); Yin et al. (2019); Zhao et al. (2020)
NaB	Histone deacetylase inhibitor	Xu et al. (2019)
PD0325901	MEK1/2 inhibitor	Yang et al. (2019)
Pifithrin-α	p53 inhibitor	Dai et al. (2015)
Purmorphamine	Smoothened agonist	Zhang et al. (2015); Qin et al. (2018); Yang et al. (2019)
P7C3	Targets NAMPT enzyme	Yang et al. (2019)
QVD-OPH	Caspase inhibitor	Liu et al. (2020)
Repsox	TGF-beta RI (ALK5) inhibitor	Cheng et al. (2015b); Hu et al. (2015); Gao et al. (2017); Oh et al. (2017); Wan et al. (2018); Yang et al. (2020c); Zhao et al. (2020)
Retinoic acid	RA receptors ligand	Qin et al. (2018)
RG108	DNA methyltransferase inhibitor	Yang et al. (2019)
SAG	Smoothened agonist	Zhang et al. (2015)
SB431542	Inhibitors of TGF-βRI, ALK4, and ALK7	Li et al. (2015); Zhang et al. (2015); Gao et al. (2019); Hu et al. (2019); Yin et al. (2019); Zhao et al. (2020)
SP600125	JNK inhibitor	Hu et al. (2015); Qin et al. (2018); Hu et al. (2019); Yang et al. (2020c)
Thiazovivin	ROCK inhibitor	Zhang et al. (2015)
TTNPB	RAR ligand	Zhang et al. (2015); Xu et al. (2019)
Vitamin C	antioxidant	Liu et al. (2020)
VPA	HDAC inhibitor	Cheng et al. (2015b); Hu et al. (2015); Zhang et al. (2015); Gao et al. (2017); Wan et al. (2018); Gao et al. (2019); Hu et al. (2019); Yang et al. (2020c); Zhao et al. (2020)
Y−27632	ROCK inhibitor	Hu et al. (2015); Li et al. (2015); Qin et al. (2018); Wan et al. (2018); Gao et al. (2019); Hu et al. (2019); Yang et al. (2019); Yang et al. (2020c); Zhao et al. (2020)

## 2 Mechanisms of Small Molecules Induced Reprogramming

The patterns of small-molecule cocktails in neuronal reprogramming are decided on the types of starting cells and whether it is under pathological conditions (Hu et al., 2015; Li et al., 2019; Qin et al., 2020). However, the synergistic mechanisms of the used drug cocktails, remain unclear (Vasan et al., 2021). It has been reported that several combinations of small molecules, such as DFICBY (DBcAMP, Forskolin, ISX9, CHIR99021, I-BET151, Y-27632) (Ma et al., 2021), CAYTFVB (CHIR99021, A8301, Y27632, TTNPB, Forskolin, Valproic acid (VPA), NaB) (Xu et al., 2019), FRSCGYIB (Forskolin, RepSox, SP600125, CHIR99021, Go6983, Y27632, IXS9, I-BET151) (Yang et al., 2020c), and VCRFSGYIBRTQ (VPA, CHIR99021, Repsox, Forskolin, SP600625, GO6983, Y27632, ISX9, I-BET151, RA, Vit C, QVD-OPH) (Liu et al., 2020), are used to modulate signaling pathways, epigenetics, and the metabolisms of reprogramming, to effectively transform somatic cells into neurons, while these researchers did not illuminate the comprehensive mechanisms. Noteworthy, challenges exist in exploring the best combinations of small molecules in converting neurons from other differentiated cells.

### 2.1 Signaling Pathways Involved in Small Molecules Induced Reprogramming

#### 2.1.1 The Wnt/GSK-3 Signaling Pathway

**FIGURE 2:**
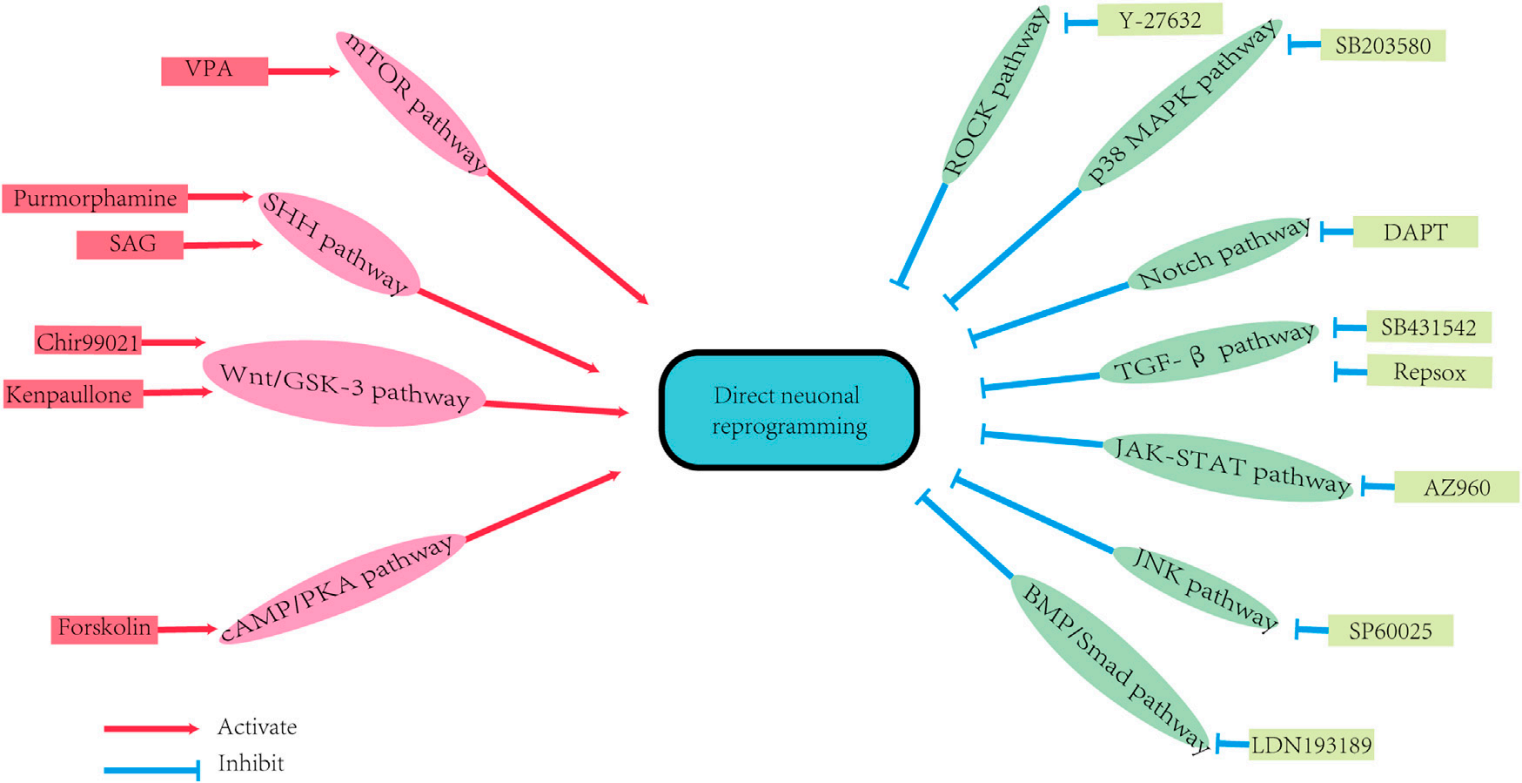
The signaling pathways of direct neuron reprogramming by small molecules. DAPT: N-[N-(3,5-difluorophenacetyl)-L-alanyl]-S-phenylglycine t-butyl ester; SAG: SHH activator smoothened agonist; VPA: Valproic acid. BMP/Smad pathway: bone morphogenetic protein/Smad pathway; cAMP/PKA pathway: cyclic adenosine monophosphate/protein kinase A system pathway; JAK-STAT pathway: Janus kinase-signal transducer and activator of transcription pathway; JNK pathway: Jun N-terminal Kinase pathway; mTOR pathway: Mammalian Target of Rapamycin pathway; P38 MAPK pathway: P38 mitogen-activated protein kinase pathway; ROCK pathway: Rho-associated protein kinase pathway; SHH pathway: Sonic Hedgehog pathway; TGF-β pathway: transforming growth factor-beta pathway; Wnt/GSK-3 pathway: (Wingless/integrated)/glycogen synthase kinase 3 pathway.

**FIGURE 3:**
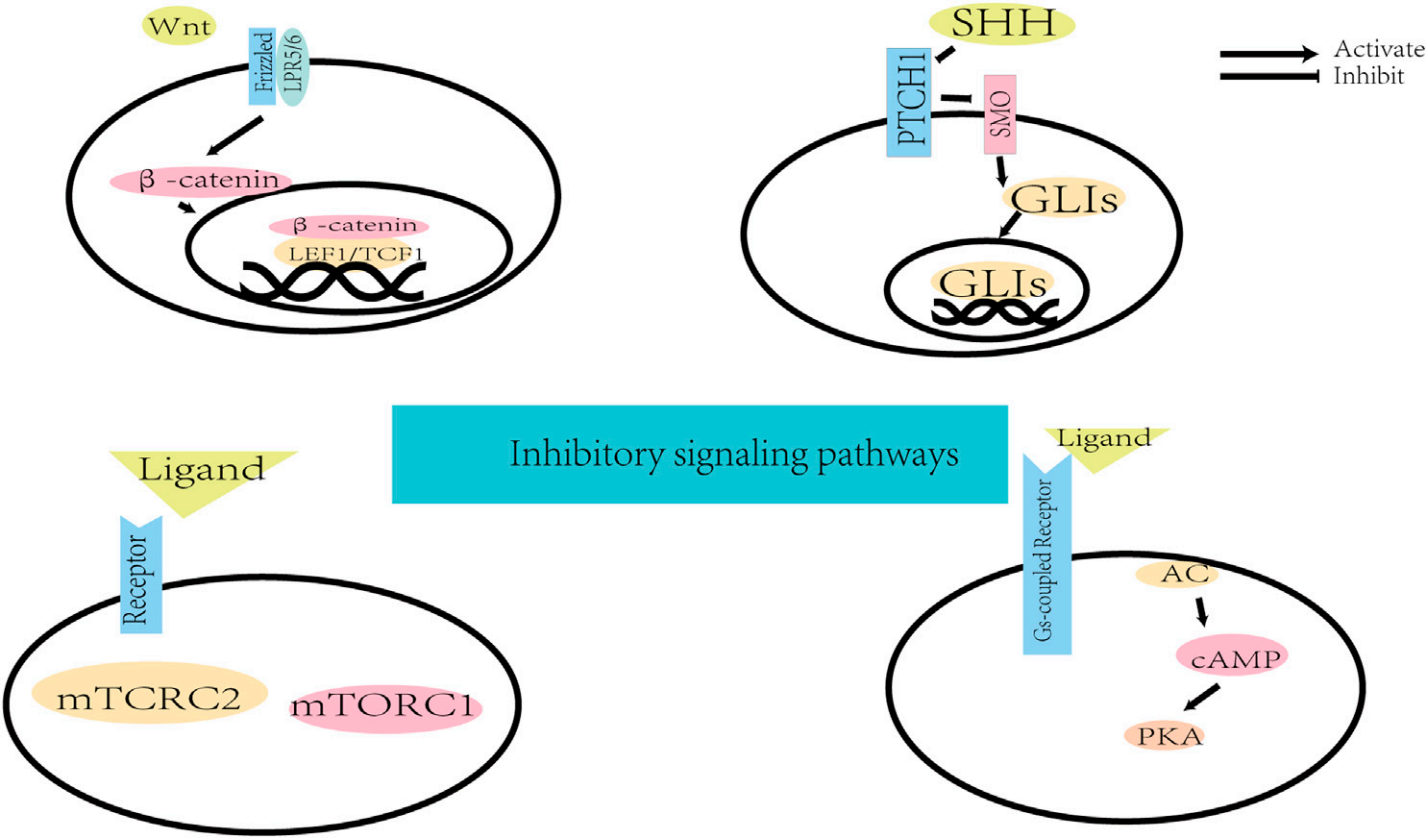
The background information of inhibitory signaling pathways.

**FIGURE 4:**
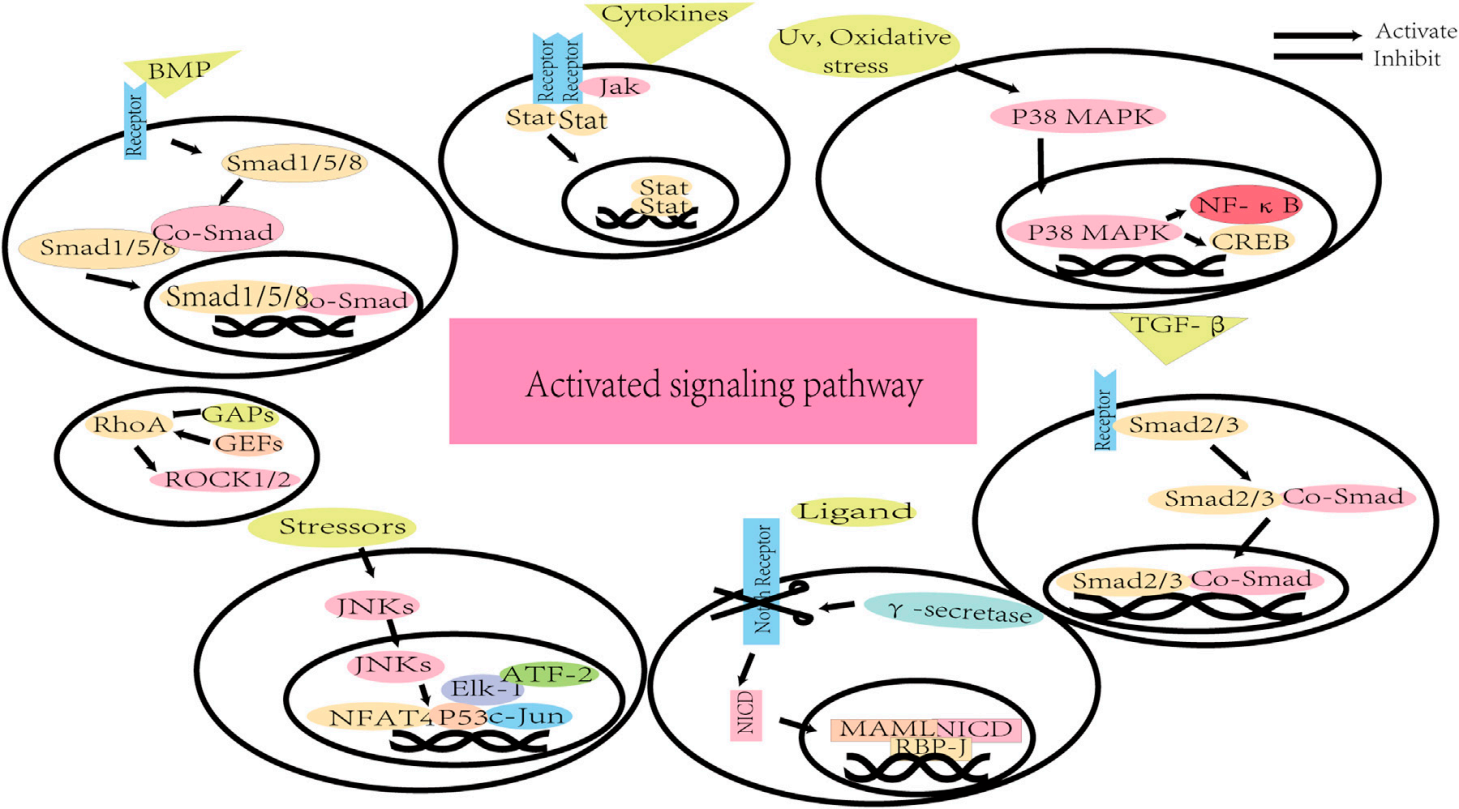
The background information of activated signaling pathways.

Small molecules can promote direct neuronal reprogramming by regulating several cellular signaling pathways (Figure 2) (Yin et al., 2019). The background information of these pathways is demonstrated in Figure 3 and Figure 4. Signaling by the Wingless/integrated (Wnt) family of secreted ligands via the transcriptional coactivator β-catenin controls embryonic development and disease processes by stimulating distinct intracellular signaling pathways (Angers and Moon, 2009; MacDonald et al., 2009). When activated by Wnt, Frizzled receptors recruit the cytoplasmic protein complex to the plasma membrane, which activates different signaling cascades (Inestrosa and Varela-Nallar, 2015). In addition, the Wnt ligands also interact with the co-receptor low-density lipoprotein receptor-related protein 5 (LRP5) or LRP6 to induce the stabilization of β-catenin by preventing its phosphorylation via glycogen synthase kinase-3 (GSK3) (Toledo and Inestrosa, 2010; Valvezan and Klein, 2012). Then β-catenin accumulates and moves to the nucleus where it interacts with members of the T-cell factor/lymphoid enhancer factor 1 (TCF/LEF1) family of transcription factors to modulate the expression of Wnt target genes (Angers and Moon, 2009; Arrázola et al., 2009; MacDonald et al., 2009; Clevers and Nusse, 2012).

The Wnt/GSK-3 signaling pathway has been shown to regulate postnatal and adult neurogenesis (Lie et al., 2005; Li et al., 2009; Zhang et al., 2011), and is also involved in the regulation of cellular self-renewal, pluripotency and promotes neuronal differentiation (Rivetti di Val Cervo et al., 2017; Oproescu et al., 2021). It has been reported that Wnt’s activation promotes the process of reprogramming (Cheng et al., 2015b). Whereas the inhibition of the GSK-3 signaling promotes neural progenitor cells’ homeostasis and induction (Hur and Zhou, 2010; Li et al., 2012). Selective GSK-3 inhibitors, such as CHIR99021 and kenpaullone (Cheng et al., 2015b), have been shown to indirectly activate the Wnt pathway in human and mouse stem cells (Silva et al., 2008; Li et al., 2009; Qin et al., 2017; Ma et al., 2019). Moreover, the combination of CHIR99021 with other small molecules has been shown to promote the conversion of astrocytes (Yang et al., 2019; Yin et al., 2019; Ma et al., 2021), fibroblasts (Qin et al., 2018), and human bone marrow mesenchymal stromal cells (MSCs)-derived neural progenitor cells (NPCs) into neurons (George et al., 2018).

#### 2.1.2 The JAK-STAT Signaling Pathway

The Janus family tyrosine kinase–signal transducer and activator of transcription (JAK-STAT) signaling pathway is broadly regulated by cytokines in CNS (Campbell, 2005). The binding of a cytokine with its unique cell membrane receptor triggers activation of the JAKs and tyrosine phosphorylation (Nabavi et al., 2019). Then the receptor phosphotyrosine sites interact with Src homology 2 (SH2) domains on STATs, and STATs also become phosphorylated by JAKs (Banerjee et al., 2017). These activated STATs form dimers that translocate to the nucleus and bind with DNA target sequences modulating gene expression (Campbell, 2005; Stark and Darnell, 2012; Au-Yeung et al., 2013).

The JAK-STAT signaling pathway is involved in cell proliferation, differentiation, apoptosis, and immune system regulation (Xin et al., 2020; Herrera and Bach, 2019; Villarino et al., 2017; O'Shea et al., 2015). Unlike neurons, which have a non-dividing property, fibroblasts and astrocytes can divide and proliferate. However, exiting the cell cycle is required for the fibroblasts and astrocytes transformation into neurons (Liu et al., 2013; Jiang et al., 2015; Tang, 2017). Interestingly, the inhibition of the JAK-STAT pathway can promote neuronal reprogramming via inducing fibroblasts to exit the cell cycle (Herdy et al., 2019). It has been demonstrated that the JAK2 inhibitor, AZ960, can reduce fibroblasts division while mediating the conversion of fibroblasts to neurons (Herdy et al., 2019).

Epithelial-mesenchymal transition (EMT) refers to a process in which epithelial cells lose their apical-basal polarity and cell-cell junctions to transdifferentiate into mesenchymal cells that have invasive properties (Amack, 2021). Mesenchymal-to-Epithelial Transition (MET) is the opposite process of EMT and has been reported to play an important role in the direct conversion of fibroblasts into neurons, and therefore, MET may be used to improve the process of reprogramming (Li et al., 2010; Samavarchi-Tehrani et al., 2010; He et al., 2017). However, the p-STAT3 dimer directly promotes the expression of genes related to EMT (Wendt et al., 2014). The JAK2 inhibitor, AZ960, has been reported to promote MET via indirect inhibition of STAT3, which leads to an enhanced conversion of fibroblasts into neurons (Herdy et al., 2019). In addition, the JAK/STAT pathway inhibitor, ruxolitinib, has been shown to improve the efficiency of cortical astroglia conversion into neurons in rats (Smith et al., 2016).

#### 2.1.3 The SHH Signaling Pathway

The founding member of the Sonic Hedgehog (SHH) family of secreted proteins three decades ago opened a field that has encompassed embryonic development, stem cell biology, and tissue homeostasis (Briscoe and Thérond, 2013). The SHH signaling has involved in axonal guidance and stem cell maintenance in the nervous system (Fuccillo et al., 2006). The SHH, released by the cell producing the ligands, relieves the constitutive repression of the receptor smoothened (SMO) by the receptor patched 1 (PTCH1) (Tukachinsky et al., 2016; Hill et al., 2021). Once SMO activation, the zinc finger transcription factors known as GLIs travel to the nucleus and initiate transcription of downstream SHH target genes (Bai et al., 2004; Pan et al., 2006; Hill et al., 2021).

Apart from regulating neural development (De Luca et al., 2016; Patel et al., 2017; Hill et al., 2021), the SHH signaling pathway can also promote the proliferation of reactive astrocytes (Sirko et al., 2013; Guo et al., 2014). Embryonic stem cells or induced pluripotent stem cells can differentiate into motor neurons after treating with the SHH/Purmorphamine (an activator of the SHH signaling pathway), indicating that activation of the SHH signaling facilitates motor neuron generation (Qin et al., 2018). The activation of the SHH pathway has been shown to promote neuronal differentiation in the transformation process of somatic cells (Masai et al., 2005). The SHH activator, smoothened agonist (SAG), can promote the *in vitro* reprogramming of human fetal astrocytes into functional neurons (Zhang et al., 2015).

#### 2.1.4 The BMP/Smad Signaling Pathway

The bone morphogenetic protein (BMP) belongs to the transforming growth factor-beta (TGF-β) superfamily and it has been demonstrated that BMPs are involved in the regulation of cell proliferation, survival, differentiation and apoptosis (Shi and Massagué, 2003; Xiao et al., 2007). When BMP ligands bind to the BMP receptors, the canonical BMP signaling pathway is initiated through activating Smads 1, 5, and 8 (Zhong and Zou, 2014; Kashima and Hata, 2018). Then the receptor-activated Smad proteins (R-Smads) form the complex with the common mediator-Smad (co-Smad, Smad4), followed by traveling to the nucleus and modulating gene expression (Heldin et al., 1997; Xiao et al., 2007).

The BMP/Smad signaling pathway regulates synaptic development and astroglial differentiation (Gross et al., 1996; Politano et al., 2019; Vuilleumier et al., 2019). Blocking BMP signaling has been shown to further promote iNs maturation (Vignoles et al., 2019). The BMP inhibitor, LDN193189, which was important for astroglial differentiation, promoted the reprogramming of human astrocytes into neurons (Gross et al., 1996; Zhang et al., 2015; Ma et al., 2019; Yin et al., 2019). For higher reprogramming efficiency, LDN193189 has been combined with SB431542 (a TGF-β inhibitor) to assist in neural transition (Lee et al., 2015; Mirakhori et al., 2015; Park et al., 2017). In addition, another BMP pathway blocker dorsomorphin and SB431542, improve the efficiency of the induction of the human adult peripheral blood T cells to iNs (Tanabe et al., 2018).

#### 2.1.5 The TGF-β Signaling Pathway

The TGF-β pathway is involved in various biological processes during embryogenesis and in adult tissue homeostases, such as cell growth, differentiation, migration, and apoptosis (Massagué et al., 2000; Derynck and Akhurst, 2007; Kashima and Hata, 2018). Ligands bind to TGF-β ligand-specific receptors with serine/threonine kinases and then receptors are activated, which activate Smad proteins 2 and 3 (Massagué et al., 2000; Dijke and Hill, 2004). The activated Smads bind co-Smad to form the complex translocates to the nucleus and modulates gene transcription by binding to DNA-binding transcription factors (Dijke and Hill, 2004; Kashima and Hata, 2018).

It has been reported that the activation of the TGF-β signaling pathway can inhibit neuronal fate and promote glial fate (Rodriguez-Martinez and Velasco, 2012). The TGF-β signaling pathway has been shown to induce EMT, and its inhibition can facilitate reprogramming by promoting MET (Aguirre et al., 2010; Qin et al., 2017). SB431542 (a TGF-β inhibitor) is involved in inhibiting glial fate and promoting neuronal fate, which not only boosts reprogramming during MET (Ichida et al., 2009; Li et al., 2010; Rodriguez-Martinez and Velasco, 2012; Zhang et al., 2015), but also promotes the cell cycle exit and conversion of human astrocytes into neurons (Rodriguez-Martinez and Velasco, 2012; Ma et al., 2019; Yin et al., 2019). In addition, SB431542 can also inhibit Smad signaling by regulating the phosphorylation of ALK (Activin-receptor-like kinase) receptors (Dijke and Hill, 2004). Recently, another common TGF-β signaling pathway inhibitor, RepSox, showed similar promoting functions in neuronal reprogramming (Tu et al., 2019; Roudnicky et al., 2020; Zhang et al., 2020; Chen et al., 2021).

#### 2.1.6 The Notch Signaling Pathway

Notch is not only a key regulator of adult neural stem cells but also plays important role in the regulation of migration, morphology, synaptic plasticity, and survival of neurons (Ables et al., 2011). Mammals possess four Notch receptors (Notch1, Notch2, Notch3 and Notch4) and many ligands, mainly containing jagged 1 (JAG1) and jagged 2 (JAG2, homologs of serrate), and delta-like proteins (Lindsell et al., 1996; Ables et al., 2011). Upon ligand binding, the transmembrane domain of the Notch receptor is cleaved by γ-secretase, and the Notch intracellular domain (NICD) is released into the cytoplasm (Ables et al., 2011). Then NICD translocates to the nucleus and is combined with mastermind-like protein 1 (MAML1), MAML2 or MAML3, which converts the recombining binding protein suppressor of hairless (RBPJ) complex from a transcriptional inhibitor to an activator to modulate Notch target genes expression (Ables et al., 2011).

The Notch signaling pathway is a highly conserved morphogenetic pathway, which plays an important role in regulating neuronal self-renewal and differentiation (Breunig et al., 2007; Roese-Koerner et al., 2017), survival (Saura et al., 2004), and neuronal plasticity (de Bivort et al., 2009; Zhang et al., 2014). The activation of this pathway maintains cells in an undifferentiated state, while its inhibition helps promote neuronal fate determination (Kageyama et al., 2005). DAPT (N-[N-(3,5-difluorophenacetyl)-L-alanyl]-S-phenylglycine t-butyl ester) inhibits Notch signaling pathway by targeting γ-secretase (UrbÃ¡n and Guillemot, 2014; Borghese et al., 2010; Hitoshi et al., 2004), and has been used to promote the neuronal differentiation of embryonic stem cells (Borghese et al., 2010; Qi et al., 2017). Besides, DAPT facilitates the conversion of human astrocytes into neurons *in vitro* (Zhang et al., 2015; Yin et al., 2019). In a mouse stroke model, the inhibition of Notch signaling is required for the entry of mouse striatal astrocytes into neurogenesis (Magnusson et al., 2014), suggesting that the conversion of reactive astrocytes into neurons is associated with Notch signaling (Smith et al., 2016).

#### 2.1.7 The p38 MAPK Signaling Pathway

MAPKs are a family of highly conserved proteins and are involved in different cellular processes including cell survival, proliferation, differentiation, and migration (Zarubin and Han, 2005; Ijomone et al., 2021). There are three major subfamilies of MAPK proteins: extracellular signal-regulated kinases (ERK), Jun N-terminal Kinase (JNK), and mitogen-activated protein kinase (p38 MAPK) (Zarubin and Han, 2005; Koga et al., 2019; Aluko et al., 2021). The p38 pathway comprises p38α, p38β, p38γ, and p38δ, which is activated by stress and regulates immune response, cell survival, and differentiation (Nebreda and Porras, 2000; Ono and Han, 2000; Cuenda and Rousseau, 2007). The MAPK pathways consist of three continuously activated protein kinases: MAPK, MAPK1, and MAPK2 (Morrison, 2012). The specific extracellular stimuli activate MAPK2 and MAPK1 successively and then initiate the p38 MAPK cascade (Ijomone et al., 2021). After activation, p38 translocates from the cytoplasm to the nucleus to modulate factors like cyclic AMP-responsive element-binding protein (CREB) and nuclear factor kappa B (NF-κB), or accumulates in the cytoplasm to regulate proteins (Raingeaud et al., 1995; Ben-Levy et al., 1998).

The activation of the p38 MAPK signaling pathway has been shown to promote astrocytes activation and glial scar formation during reactive gliosis (Cheon et al., 2016; Li et al., 2020). Its inhibition improved neuronal induction by inhibiting the proliferation of responsive astrocytes. SB203580 is a common p38 inhibitor that has been applied to induce the conversion of reactive astrocytes to neurons *in vitro* (Smith et al., 2016).

#### 2.1.8 The mTOR Signaling Pathway

The mechanism of the Mammalian Target of Rapamycin (mTOR) signaling pathway is a crucial cellular signaling hub, involving growth control, protein synthesis, gene expression, and metabolic regulation (Lipton and Sahin, 2014). The mTOR is a large, highly conserved, serine/threonine kinase, which is a member of the phosphoinositide 3-kinase-related kinase family and is widely expressed in eukaryotic cell types, including neuronal cells (Sabatini et al., 1999). Extracellular activators of the mTOR pathway bind to membrane receptors, and then trigger intracellular signal transduction via mTOR complex 1 (mTORC1) and mTOR complex 1 (mTORC2) (Lipton and Sahin, 2014; O' Neill, 2013). The activation of the mTOR pathway regulates protein syntheses and enhances gene expressions involved in the regulation of cell proliferation and survival (Russo et al., 2012).

Studies have reported that the mTOR signaling pathway is a critical regulator of cell growth and proliferation (Endo et al., 2009; Kaeberlein, 2013; Qin et al., 2016). The reprogramming efficiency and neuronal differentiation may be enhanced by the VPA (an activator of the mTOR pathway)-activated mTOR signaling pathway (Duan et al., 2019).

#### 2.1.9 The cAMP/PKA Signaling Pathway

The cAMP is the archetypal second messenger discovered in 1956 (Sutherland and Rall, 1958). A high level of cyclic adenosine monophosphate (cAMP) is connected with axonal growth during the development of the embryonic CNS (Cai et al., 2001). When an extracellular ligand binds to its specific Gs-protein coupled receptor and induces a structural change resulting in the Gs-protein complex dissociating, the cAMP signaling pathway is initiated (Billington and Penn, 2003). Gsα subunit from Gs-protein complex drives the classical signaling pathway via activation of adenylyl cyclase (AC) (Billington and Hall, 2012). Then AC catalyzes the conversion of intracellular ATP to cAMP. As the downstream of cAMP, the protein kinase A system (PKA) is activated and modulates protein phosphorylation and gene expression (Billington and Hall, 2012; Yang, 2018).

The cAMP is a ubiquitous second messenger in the regulation of many biological processes, such as cell migration, differentiation, proliferation, and apoptosis (Zhang et al., 2016b). Forskolin is a cAMP/PKA activator that stimulates the transformation of fibroblasts into neurons (Liu et al., 2013; Hu et al., 2019; Cardon et al., 2020; Yang et al., 2020c). Furthermore, the increased intracellular levels of cAMP by Forskolin provide neurotrophic effects, reduce oxidant stress injury, and improve cellular survivability (Xu et al., 2019). It has also been found that small molecules that target the cAMP/PKA pathway can overcome the interference of *in vivo* environment on transdifferentiation (Yuan et al., 2020).

#### 2.1.10 The ROCK Signaling Pathway

The Rho-associated protein kinase (ROCK) is a serine-threonine kinase with two isoforms (ROCK1 and ROCK2), the inhibition of which causes several biological events such as promotion of neurite outgrowth, axonal regeneration, and activation of prosurvival Akt (Nakagawa et al., 1996; Chong et al., 2017). As the upstream of the ROCK, RhoA (a small GTPase protein) is regulated by guanine nucleotide-exchange factors (GEFs) and GTPase-activating proteins (GAPs) to shift its activation or inactivation (Nakagawa et al., 1996; Cherfils and Zeghouf, 2013; Duman et al., 2015). The activated RhoA activates the ROCK1 or ROCK2, followed by driving actin cytoskeletal remodeling, cell contractility, and cell death (Leung et al., 1995; Matsui et al., 1996; Kimura et al., 2021).

The ROCK signaling pathway is closely associated with the pathogenesis of various CNS disorders through its involvement in many aspects of neuronal functions, including neurite outgrowth and retraction (Koch et al., 2018). The inhibitor of ROCK, Thiazovivin, has been shown to improve cell survival and promote reprogramming efficiency (Watanabe et al., 2007; Lin et al., 2009). Another inhibitor of the ROCK pathway, Y-27632, helps induce fibroblasts into neuron-like cells (Hu et al., 2015). In addition, a study showed that Y-27632 could also effectively reduce reactive oxygen species (ROS) levels in fibroblasts during the process of motor neuron regeneration after injury (Kang et al., 2017).

#### 2.1.11 The JNK Signaling Pathway

The JNK is one of the subfamilies of MAPK, accumulating evidence indicating that this family is also important for neuronal migration during brain development (Sun et al., 2007). Following exposure stressors, the JNKs translocate from the cytoplasm to the nucleus (Zhang et al., 1998). In the nucleus, JNKs phosphorylate several transcription factors, such as c-Jun, ATF-2, Elk-1, p53, and NFAT4, which then trigger specific cell stress responses (Tournier et al., 2000; Sun et al., 2007).

The JNK pathway is an evolutionarily conserved kinase cascade, which plays an important role in stress-induced apoptosis and tumor progression (Herrera and Bach, 2021). It is widely confirmed that there is oxidative stress during direct reprogramming (Gascón et al., 2016; Suzuki and Shults, 2019), and therefore, the inhibition of the JNK pathway can improve the survival of iNs. In this case, the JNK inhibitor, SP600125, is combined with other small molecules to drive the transdifferentiation of somatic cells into neurons (Hu et al., 2015; Hu et al., 2019; Liu et al., 2020).

Since each small molecule activates or inhibits different signaling pathways, the use of different drug cocktails results in variable neuronal properties and conversion efficiencies. It is challenging to elucidate the exact role of each chemical compound reprogramming. Furthermore, only pan-signaling pathways that are regulated by small molecules, are proposed. The specific targets of the chemical during transdifferentiation remain unclear and need further investigations. The review of published studies indicates that various combinations of small molecules are used for neuronal reprogramming. Notably, despite the analogs that act on the same pathway, the individual effect of each small molecule may not be consistent, resulting in different conversion efficiencies. For example, a study demonstrated that when replacing the cocktails, SLCD, consisting of SB431542 (S), LDN193189 (L), CHIR99021 (C), and DAPT (D) by their functional analogs in astrocytes conversion into neurons, the conversion efficiency becomes lower than that of the original SLCD combination (Yang et al., 2020c). Besides, the molecular pathways involved in the transformation process vary, due to the different characteristics of the starting cells, and the complexity of the pathways’ network. Therefore, it is complicated to interpret the specific mechanisms of these molecular combinations.

### 2.2 The Modulation of Epigenetics by Small Molecules

Some small molecules can regulate the efficiency of reprogramming by modulating epigenetics (Aguirre-Vázquez et al., 2021). These epigenetic modifications are heritable differences that include “Tags” such as DNA methylation and diverse histone modifications that can affect DNA accessibility and chromatin structure (Ebrahimi et al., 2019). DNA methylation and histone modifications are the main mechanisms responsible for the epigenetic regulation of gene expression during cell development and differentiation (Li et al., 2007; Cedar and Bergman, 2009). In general, DNA methylation is associated with gene silencing (Bird, 2002). DNA methyltransferases (DNMTs) are a family of enzymes responsible for DNA methylation (Tessier et al., 2021). DNMTs inhibitors, such as 5-aza-29-deoxycytosine (5-aza-dC), and RG108, have been shown to promote neuronal reprogramming by increasing DNA methylation of non-neuronal gene promoters (Zhang et al., 2015; Zeng et al., 2021). Zhang et al. revealed a significant increase of DNA methylation in the promoter region of the GFAP (glial fibrillary acidic protein) gene *via* epigenetic analyses during the chemical reprogramming, consistent with previous studies on epigenetic alteration of astrocytes during the neuronal reprogramming (Condorelli et al., 1994; Cheng et al., 2011; Zhang et al., 2015). Histones can be post-translationally modified via methylation and acetylation. Histone methylation contributes to gene activation or repression, while histone acetylation is likely to correlate with gene activation (Struhl, 1998; Boyer et al., 2006; Barski et al., 2007; Kouzarides, 2007; Smith et al., 2016). Histone acetylation is beneficial to open chromatin structures through its disruption of nucleosomal interactions and histone tails’ release from the linker DNA (Kretsovali et al., 2012), which is the basis for the binding of chromatin by neural TFs that facilitate the activation of neuronal programs (Smith et al., 2016). For example, H3K9 (histone H3 at lysine 9) acetylation is one of the most momentous epigenetic markers. This acetylation process can promote gene expression in transdifferentiation when it is enriched in the promoter region of genes (Liang et al., 2004; Bernstein et al., 2005). H3K27 (histone H3 at lysine 27) acetylation is perceived as a super-enhancer that promotes gene expression (Hnisz et al., 2013). Enrichment in H3K27 acetylation has been observed at TF binding sites in reprogrammed fibroblasts into neurons (Smith et al., 2016). The increased histone acetylation level by the HDACs (histone deacetylases) inhibitor, VPA, has been confirmed to increase the level of H3K4 methylation in the promoter region and decrease the level of H3K27 methylation at the transcription start site (TSS) of the NEUROG2 and NEUROD1, which leads to an improved reprogramming efficiency (Phiel et al., 2001; Huangfu et al., 2008; Shi et al., 2008; Hirabayashi et al., 2009; Zhang et al., 2015; Qin et al., 2017; Yin et al., 2019). However, VPA did not appear to serve a notable role or even decreased the reprogramming efficiency in several chemical cocktails (Yin et al., 2019; Yang et al., 2020c). An exciting finding showed that more cell death was observed after longer exposure to VPA because of its cytotoxicity (Zhang et al., 2015). In addition, all-trans retinoic acid (ATRA) can activate histone acetyltransferases (HAT) by binding retinoic acid (RA) receptors to improve the conversion of neuronal transdifferentiation (Han et al., 2010). TTNPB (an agonist of RA receptors) was found to play an important role in neural differentiation, while did not have a significant effect on the astrocyte-neuron reprogramming (Zhang et al., 2015). The lack of contribution of TTNPB indicated that RA may not be a necessary element in reprogramming astrocytes into neurons. Administration time and dosage of epigenetic regulators should be explored when incorporated into chemical cocktail formulas. Moreover, some small molecules, such as forskolin and dorsomorphin (inhibitor of AMP-activated protein kinase and BMP type 1 receptors), have been shown to induce reprogramming, by modulating the epigenetic state, improving chromatin accessibility, and promoting H3K27 acetylation (Liu et al., 2013).

At present, research on the regulation of epigenetics by small molecules is a novel and promising direction in the mechanistic studies of reprogramming. The modulation of epigenetics is temporal and reversible to the unchangeable DNA sequence of the host cells, which is retained during the disease state and age information of the starting cells. Some new techniques, such as high-throughput sequencing analysis, can be utilized to screen pivotal regulators of epigenetics (Headley et al., 2019; Vandana et al., 2021).

### 2.3 Modulation of Cellular Metabolism by Small Molecules

The metabolic state of cells is affected by intracellular and extracellular factors, such as specific cell cycle phases and cell functions, the oxygenation status, and metabolic demands of tissues under different physiological or pathological conditions (Ito and Suda, 2014). In general, proliferating cells, such as astrocytes and fibroblasts, mainly use anaerobic glycolysis and beta-oxidation for energy metabolism (Lunt and Vander Heiden, 2011; Ge et al., 2012; Gascón et al., 2017; Qin et al., 2017), whereas neurons are more dependent on oxidative phosphorylation (OXPHOS) (Tsacopoulos and Magistretti, 1996; Hülsmann et al., 2000; Bélanger et al., 2011; Shyh-Chang et al., 2013; Magistretti and Allaman, 2015; Zheng et al., 2016), which increase is a characteristic of neuronal identity. Some studies reported that anaerobic glycolysis limits neuronal reprogramming (Agostini et al., 2016; Gascón et al., 2016; Zheng et al., 2016). During the conversion of astrocytes into neurons, a downregulation of glycolysis-related genes and an increase in OXPHOS-related genes have been observed (Magistretti and Allaman, 2015; Masserdotti et al., 2016; Gascón et al., 2017; Ma et al., 2019). Hypoxia-inducible factor 1-alpha (HIF-1α) is a major inhibitor of OXPHOS and an activating mediator of glycolysis. The HIF-1α inhibitor, KC7F2, can improve the induction of neuronal conversion by promoting OXPHOS and its metabolic shift which are necessary for successful induction of neuronal conversion (Herdy et al., 2019). In addition to metabolic state, the metabolic factor ROS can also influence the regulation of cell fate (Maryanovich and Gross, 2013). During neuronal reprogramming, metabolic shift causes oxidative stress, which is a major obstacle to successful conversion (Gascón et al., 2016). It was also reported that there is an increase in lipid peroxidation during the reprogramming of astrocytes into neurons, and that antioxidants, such as vitamin E and forskolin, can promote iNs generation by reducing lipid peroxidation (Liu et al., 2013; Gascón et al., 2016).

In summary, the process of direct neuronal reprogramming is often accompanied by a metabolic shift that leads to an increase in oxidative products, which may inhibit the reprogramming process, and lead to cell death. Therefore, small molecules that can promote metabolic shift and inhibit oxidative stress may be needed in improving the efficiency of reprogramming.

## 3 Methods to Screen Small Molecule Cocktails

There are several approaches to screening small molecule compounds in the process of reprogramming. Yang et al. screened small molecules based on three major criteria of selection: 1) the disruption of somatic cell-specific programs, such as I-BET151 (Li et al., 2015); 2) the activation of neural reprogramming related signaling pathways, the promotion of neuronal gene expression, and the improvement of reprogramming efficiency, such as VPA, Forskolin, CHIR99021, GO6983, SP600125, ISX9, purmorphamine and dorsomorphin (Huangfu et al., 2008; Ladewig et al., 2012; Hu et al., 2015; Li et al., 2015; Madhu et al., 2016); and 3) the potential to promote neuronal survival and maturation, such as Repsox and Y-27632 (Ladewig et al., 2012; Lamas et al., 2014; Li et al., 2015). In addition, a team identified compounds that predominantly target epigenetic modifications, metabolic transformations, and signaling pathways involved in neuronal fate patterning (Yang et al., 2019). Moreover, Li et al. have used a stepwise protocol in which they screened for small molecules that improved the efficiency of transcription-factor-mediated reprogramming and determined if a combination of the resulting molecules could completely replace the involved TFs (Vierbuchen et al., 2010; Li et al., 2015). The authors found that combining small molecules which drive the conversion of somatic cells into neural progenitor cells, with small molecules that boost the differentiation of the neural progenitors to neurons, directly induces neuronal reprogramming (Hu et al., 2015). In 2016, another study showed that an unbiased screening assay can be used to screen small molecules to effectively increase the efficiency of reprogramming fibroblasts into neurons (Yin et al., 2019). Although numerous, current studies lack effective and standard methods to screen optimal small-molecule cocktail formulas. As we all know, there are plentiful small molecules with similar functions, which makes it challenging to choose the best formulas under different conditions. Theoretically, the above methods may be feasible for directly neuronal reprogramming, but extensive repetitions of experiments are still needed to illustrate these protocols.

When somatic cells are cultured in mediums until they reach a 90% confluence, treatment with small-molecule cocktails can initiate reprogramming (Zhang et al., 2015; Qin et al., 2018; Yin et al., 2019). Direct small molecules-mediated reprogramming is generally divided into two steps: One is associated with the induction culture, which is mainly mediated by small molecules; and the other is the differentiation or maturation culture, which is simply maintained by neurotrophic factors or fewer small molecules (Li et al., 2015; Gao et al., 2017; Ma et al., 2019; Yang et al., 2019). Sometimes, the newly generated neurons are also co-cultured with primary glial cells to support the maturation of induced neuronal electrophysiology and synaptic activities, indicating that glial-derived factors may contribute to the reprogramming (Kashani et al., 2011; Gao et al., 2017; An et al., 2018).

## 4 Chemical Cocktail Formulas in the Transdifferentiation of Somatic Cells Into Neurons

**FIGURE 5:**
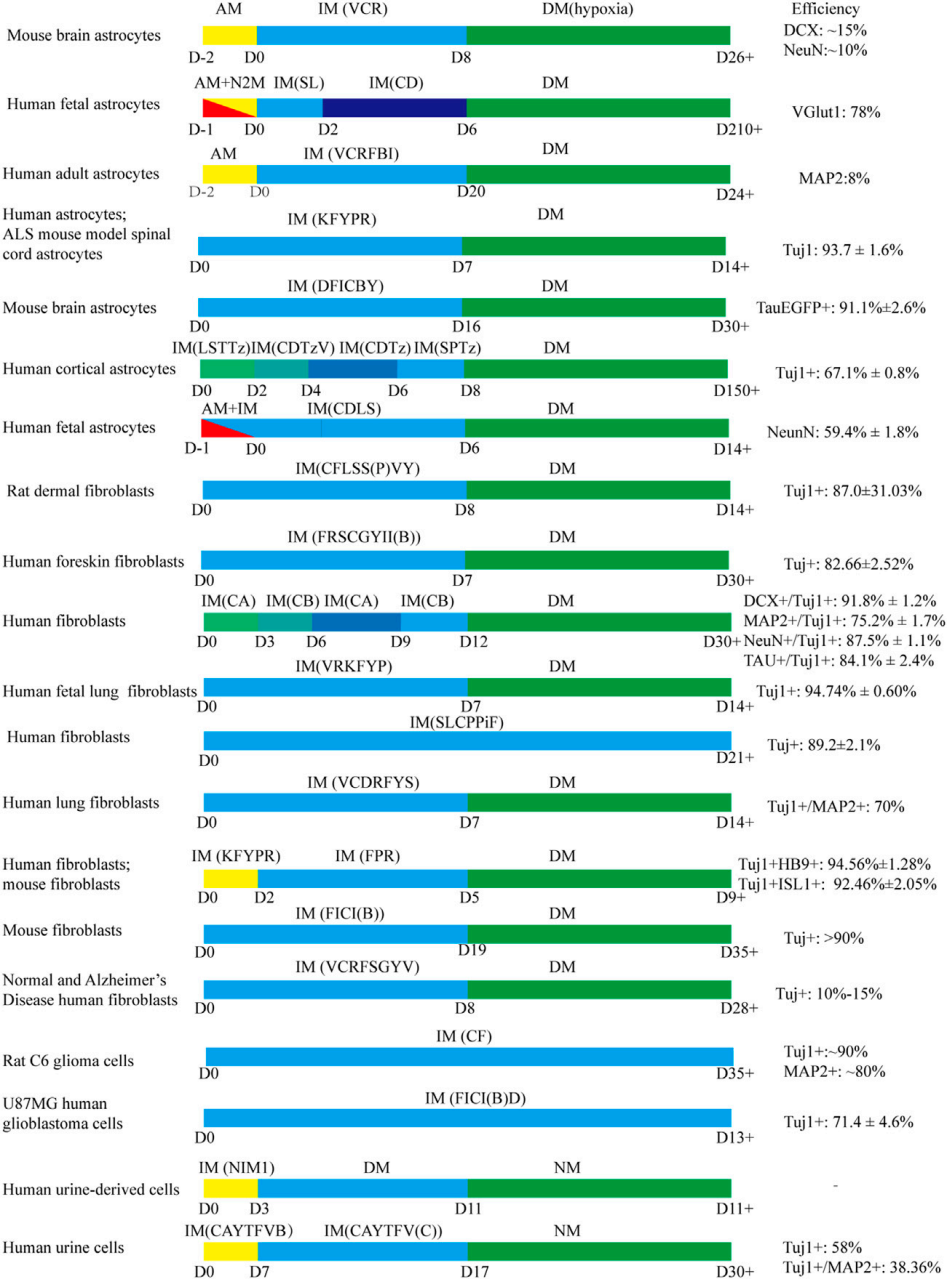
The common protocols use small molecules to generate iNs. AM: astrocyte medium; IM: induced medium; DM: differentiation medium; N2M: N2 medium; NM: neuron medium VCR: VPA, Chir99021, Repsox; SL: SB431542, LDN193189; CD: CHIR99021, DAPT; VCRFBI: VPA, Chir99021, Repsox, Forskolin, i-Bet151, ISX-9; KFYPR: Kenpaullone, Forskolin, Y-27632, Purmorphaine, Retinoic acid; DFICBY: DBcAMP, Forskolin, ISX9, CHIR99021, IBET151, Y-27632; LSTTz: LDN193189, SB431542, TTNPB, Tzv; CDTzV: CHIR99021, DAPT, Tzv, VPA; CDTz: CHIR99021, DAPT, Tzv; SPTz: SAG, Purmorphamine, Tzv; CDLS: CHIR99021, DAPT, LDN193189, SB431542; CFLSS(P)VY: CHIR99021, Forskolin, LDN193189, SB431542, SP600125, VPA, Y27632; FRSCGYII(B): Forskolin, RepSox, SP600125, CHIR99021, GO6983, Y-27632, ISX-9, I-BET151; CA: CHIR99021, LDN193189, RG108, Dorsomorphin, P7C3-A20, A83-01, ISX9; CB: Forskolin, Y27632, DAPT, PD0325901, A83-01, purmorphamine, P7C3-A20; VRKFYP: VPA, Repsox, Kenpaullone, Forskolin, Y-27632, Purmorphamine; SLCPPiF: SB431542, LDN-193189, CHIR99021, PD0325901, Pifithrin-α, Forskolin; VCDRFYS: VPA, CHIR99021, DMH1, Repsox, Forskolin, Y-27632, SP600125; KFYPR: KenpaulloneKenpaullone, Forskolin, Y27632, Purmorphamine, RA; FPR: Forskolin, Purmorphamine, RA; FICI(B): Forskolin, ISX9, CHIR99021, I-BET151; VCRFSGYV: VPA, CHIR99021, Repsox, Forskolin, SP600625, GO6983, Y-27632; D, Dorsomorphin; CF: CHIR99021, Forskolin; FICI(B)D: Forskolin, ISX9, CHIR99021, I-BET151, DAPT; NIM1: cAMP-Na, VPA, CHIR99021, Repsox, Forskolin, SP600125, GO6983, Y-27632, IBET151, ISX-9, RA, QVDOPh, vitamin C; CAYTFVB: CHIR99021, A8301, Y-27632, TTNPB, Forskolin, VPA, NaB; CAYTFV(C): CHIR99021, A8301, Y-27632, TTNPB, Forskolin, vitamin C.

Small molecule cocktail formulas in the direct transformation of somatic cells into neurons were collected in Table 2. Furthermore, several common transformation protocols and conversion efficiency were summarized in Figure 5. Reviewing the published research, we speculate that the chemicals should be administrated in order. Generally, small molecules were added to induce neuronal conversion for one or 2 weeks firstly, then followed by administrating neurotrophic factors. It is hard to say which molecule is essential or optional in chemical cocktails. Various studies reached diverse conclusions due to different starting cells. Several studies reported that a combination of small chemicals, VCRF (VPA, CHIR99021, Repsox, Forskolin), had important effects on the neural differentiation and survival of mouse and human somatic cells (Cheng et al., 2015a; Hu et al., 2015; Li et al., 2015). Therefore, we speculate that VCRF can be used as a basic formula for direct neuronal reprogramming. However, it is a pity that there is no powerful evidence to confirm this. There are two most common types of somatic cells used for neuronal reprogramming, fibroblasts and astrocytes. Therefore, we will mainly talk about the small molecule cocktail formulas in the process of transdifferentiation starting from these two types of cells.

**TABLE 2:** Small molecule cocktail formulas in direct neuronal reprogramming.

Somatic cells	Neurons	Cocktails formula	Bibliography
Human and mouse fibroblasts	Motor neurons	FK(e)P(u)R(e)Y(5)	Qin et al. (2018)
Human Fetal Astrocytes	Glutamatergic, GABAergic dopaminergic neurons	CDLS(B) (4)	Yin et al. (2019)
Human lung fibroblasts	Neurons	CD(M)FRS(P)V(P)Y(7)	Wan et al. (2018)
Human urine-derived cells	GABAergic neurons	CFGII(S)QRR(e)S(P)VV(P)Y(12)	Liu et al. (2020)
Adult human retinal pigment epithelial cells	Dopaminergic neuron	CLS(B) (3)	Li et al. (2019)
Mouse astrocytes	Dopaminergic, GABAergic, glutamatergic neurons and motor neurons	CRV(P) (3)	Cheng et al. (2015b)
IMR−90 fibroblasts	Dopaminergic neuron-like cells	FK(e)P(u)RV(P)Y(6)	Qin et al. (2020)
Human urine cells	Glutamatergic neurons	ACFNT(T)V(P)Y(7)	Xu et al. (2019)
Normal and Alzheimer’s disease human fibroblasts	Glutamatergic neurons	CFGRS(P)V(P)Y(7)	Hu et al. (2015)
Adult astrocytes	Glutamatergic neurons	CFII(S)RV(P) (6)	Gao et al. (2017)
Glioblastoma Cells	Neurons	CDFII(S) (5)	Lee et al. (2018)
Human astrocytes and ALS mouse model spinal cord astrocytes	Motor neuron-like cells	CDD(o)FK(e) (5)	Zhao et al. (2020)
Postnatal human fibroblasts	Glutamatergic, GABAergic, dopaminergic neurons	CFLPP(i)S(6)	Dai et al. (2015)
Mouse astrocytes	Glutamatergic, GABAergic neurons	CD(B)FII(S)Y(6)	Ma et al. (2021)
Human Fibroblasts	Glutamatergic GABAergic neurons	ACDD(o)FI(S)LPP(u)P(7)R(G)Y(12)	Yang et al. (2019)
C6 glioma	Neurons	CF(2)	Oh et al. (2017)
Human fibroblasts	Glutamatergic, GABAergic neurons	CFGII(S)RS(P)Y(8)	Yang et al. (2020c)
Human astroglial cells	Glutamatergic, GABAergic neurons	CDLP(u)SS(B)TT(T)V(P) (9)	Zhang et al. (2015)
Mouse fibroblasts	Glutamatergic, GABAergic neurons	CFI(S)S(B) (4)	Li et al. (2015)
Rat dermal fibroblasts	Neurons	CFLS(B)S(P)V(P)Y(7)	Hu et al. (2019)

A: A83-01; C: CHIR99021; D: DAPT; D(B): DBcAMP; D(o): Dorsomorphin.

D(M): DMH1; F: forskolin; G: GO6983; I: I-BET151; I(S): ISX-9; K: KC7F2.

K(e): Kenpaullone; L: LDN193189; N: NaB; P: PD0325901; P(i):Pifithrin-α.

P(u): purmorphamine; P(7): P7C3; Q: QVD-OPH; R: repsox; R(e): Retinoic acid; RG: RG108; S: SAG; S(B): SB431542; S(P): SP600125; T: Thiazovivin.

T(T): TTNPB; V: vitamin C; V(P): VPA; Y: Y-27632.

Fibroblasts are the most common somatic cells of neuronal reprogramming. Generally, the induction of fibroblasts derived from different species and sites or *in vivo* and *in vitro* into neurons needs diverse cocktail formulas (Hu et al., 2015; Li et al., 2015; Wan et al., 2018). However, human and mouse fibroblasts, even rat fibroblasts can be efficiently and directly converted into neurons by the same small molecule cocktails (Qin et al., 2018; Hu et al., 2019). In 2015, Li et al. found that in the cocktail, FICSB (Forskolin, ISX9, CHIR99021, SB431542, and I-BET151), the neurogenesis inducer ISX9 was necessary to activate neuron-specific genes, and SB431542 was dispensable for generating neurons, although it enhanced the survival and neurite outgrowth of the iNs. In addition, I-BET151 (a BET family bromodomain inhibitor) disrupted the fibroblast-specific programs in early-stage reprogramming, dramatically enhancing the reprogramming rate and neurite outgrowth of the iNs (Li et al., 2015). Small molecules, Repsox, Y-27632, CHIR99021, Dorsomorphin, Forskolin, Kenpaullone, LDN193189, P7C3-A20 and SP600125 have been reported to promote neuronal survival, differentiation and maturation (Rawal et al., 2007; Hotta et al., 2009; Ladewig et al., 2012; Liu et al., 2013; Lamas et al., 2014; Hu et al., 2015; Qin et al., 2018; Wan et al., 2018; Yang et al., 2019). Yang et al. claimed that RG108, PD0325901 and A83-01 supplementation boosted the neuronal reprogramming efficiency significantly (Yang et al., 2019). Interestingly, the absence of either PD03259 or A83-01 had a slight effect on the conversion rate (Yang et al., 2019). We noticed that Forskolin was present in almost all small-molecule cocktails of the existing transdifferentiation of fibroblasts into neurons, the reason for which may be due to it promoting reprogramming as a PKA activator and antioxidant, or the synergy with other chemicals.

As for astrocytes, another somatic cell widely used in the field of neuronal reprogramming, are shared the same neuroectodermal linage and the same progenitor radial glia with neurons, which may partially underlie astrocyte-to-neuron conversion (Rao et al., 2021). Cheng et al. declared that VPA alone was able to induce astrocytes into neuroblasts with low efficiency, while the elimination of VPA significantly abolished the generation of neuroblasts from postnatal mouse astrocytes (Cheng et al., 2015b). Consistent with fibroblast-to-neuron reprogramming, ISX-9 activated neuronal genes while I-BET151 suppressed astrocyte genes in the direct generation of neuronal cells from adult astrocytes (Gao et al., 2017). Kenpaullone was shown to play an important role in the neuronal morphological changes in a chemical cocktail (Zhao et al., 2020). DAPT, CHIR99021, SB431542, LDN193189, DBcAMP and Y-27632 significantly enhanced the neuronal conversion efficiency of mouse astrocytes, and DAPT was the most (Ma et al., 2021).

In addition to fibroblasts and astrocytes, human urine-derived cells, which are easily obtained without invasive injury, were converted into neuron-like cells with the help of VPA, CHIR99021, Repsox, Forskolin, SP600625, GO6983, Y27632, ISX9, I-BET151, RA, VitC, QVD-OPH and CHIR99021, A8301, Y27632, TTNPB, Forskolin, VPA, NaB (Xu et al., 2019; Liu et al., 2020). More promisingly, glioblastoma cells and glioma cells can be transdifferentiated into fully differentiated neurons by chemical cocktails hence losing their malignant characteristics (Oh et al., 2017; Lee et al., 2018).

The comprehensive roles and synergetic effects of these small molecules in neuronal conversion remain to be further explored. It is hard to say the pros and cons of different chemical cocktails. On the one hand, the neuronal transdifferentiation was inefficient and ineffective when fewer small molecules were involved (Cheng et al., 2015b). On the other hand, it has been shown that the more small molecules were contained in the formulations, the greater the toxic effect and was difficult for clinical applications (Zhang et al., 2015; Yin et al., 2019).

## 5 Detection of the Efficiency of the Reprogramming Conversion

The transformation efficiency is one of the most momentous focuses in neuronal reprogramming. There are various existing detection techniques to evaluate the conversion efficiency of neuronal reprogramming. The most significant conversion of somatic cells into neurons usually occurs within the first 24 h of treatment with small molecules (Li et al., 2015; Ma et al., 2019). Therefore, it is necessary to observe the morphology change frequently on 1 day after the administration of chemicals. An easy and direct detection method is to microscopically observe the typical morphology of the reprogrammed cells. The iNs usually have smaller, more compact, and more shining cell bodies, with bipolar or multipolar shapes, and the appearance of secondary and tertiary branches (Xu et al., 2019; Yang et al., 2019; Ma et al., 2021). The neurite length can be captured by confocal microscopy (Gao et al., 2017). In addition, the conversion of astrocytes to neurons can be dynamically monitored by time-lapse live-cell imaging or continuous photo taking *in vitro* (Zhang et al., 2015; Gascón et al., 2016; Yin et al., 2019; Rao et al., 2021); however, these methods will be technically and expensively prohibitive for most research labs (Wang et al., 2021b). Besides, immunofluorescence staining is one of the most common methods used for the detection of reprogramming efficiency. Generally, successfully induced neurons express neuronal markers, such as β-tubulin III (TUJ1), microtubule association protein-2 (MAP2), neuronal nuclei (NeuN), and doublecortin (DCX) (Rao et al., 2021). The origin of iNs can be determined by co-labeled fluorescence markers of initiating cells and neurons, which can be observed by epifluorescent microscopy or confocal microscopy *in vitro* (Xu et al., 2019; Yin et al., 2019; Ma et al., 2021). Moreover, immunostaining with synapsin I highlight the presence of strong iNs synaptic puncta along dendrites, indicating the formation of the well-established synapses (Hu et al., 2015; Yang et al., 2019).

However, the observations of morphology and immunofluorescence staining cannot identify the lineage origins of iNs which is vital for neuronal reprogramming. In 1981, Sternberg and Hamilton were the first to describe Cre, a recombinase enzyme isolated from the P1 bacteriophage, that catalyzes a directional DNA recombination between two 34-bp recognition elements called loxP sites (Sternberg and Hamilton, 1981). The Cre-loxP lineage tracing system is a common conditional lineage tracing technology that specifically labels the original cells *in vivo* (Ma et al., 2021). In this regard, the *Aldehyde dehydrogenase 1 family member L1-Cre (Aldh1l1-Cre)* Mice were intracranially injected with a flexed EGFP (Enhanced Green Fluorescent Protein)-adeno-associated virus (AAV-FLEX-EGFP), that specifically labels Aldehyde dehydrogenase 1 family member L1 (ALDH1L1)-expressing astrocytes (Ma et al., 2021). Another tracing technology is mediated by a virus vector that labels the starting cells with a green fluorescent protein (GFP) (Zhang et al., 2015; Gao et al., 2017; Yin et al., 2019), which is more specific and precise than fluorescence staining. For example, Gao and his colleagues traced the cultured astrocytes with retrovirus expressing GFP from human GFAP promoter (GFAP::GFP) (Gao et al., 2017). These starting cells’ labeling techniques can be utod in co-label with neuron-specific markers to identify the origin of iNs. In addition, a common and achievable method is to detect the expression of neuronal-specific proteins using western blot (Hu et al., 2015; Oh et al., 2017). Similarly, RT-qPCR (real-time quantitative polymerase chain reaction) can be used to monitor the expression of neuronal-specific genes (Qin et al., 2018; Yang et al., 2019).

The successful reprogramming is characterized by the iNs and endogenous neurons sharing similar transcriptional patterns. When investigating the transcriptional mechanisms of reprogramming, RT-qPCR can also be applied to quantitatively detect the differentially expressed genes (DEGs) (Qin et al., 2018; Yang et al., 2019; Yin et al., 2019). The RNA-sequencing (RNA-seq) technology is also used to investigate the transcriptome changes during the somatic cell-to-neuron conversion process (Gao et al., 2017; Ma et al., 2019; Yin et al., 2019). Comparing single-cell RNA-sequencing data from endogenous neurons and iNs a more efficient reprogramming can be achieved by correcting the differential gene expression using the Clustered Regularly Interspaced Short Palindromic Repeats (CRISPR) technique (Götz and Bocchi, 2021). The results of Gene Ontology (GO) analysis can also be used to identify similarities between iNs and endogenous neurons by determining the DGEs involved in different cell fates (Yang et al., 2019; Ma et al., 2021).

Apart from the change in cell morphology and gene expression, it is equally important that the iNs possess mature electrophysiological functions. Electrophysiological properties of iNs can usually be examined by the whole-cell patch-clamp technique that records action potentials, inward sodium currents, and outward potassium currents *in vitro* (Ma et al., 2019; Yang et al., 2019; Ma et al., 2021). The detection of typical spontaneous postsynaptic currents (sPSCs) indicates the formation of synaptic connections among neurons (Hu et al., 2015; Zhang et al., 2015; Gao et al., 2017).

Taken together, the identification of converted neurons should be validated from various aspects, such as the expression of neuron-specific genes and protein markers, and neuronal electrophysiological function, which is the basis for the application of the chemical-induced neurons (CiNs) in clinical translation.

Moreover, most important is employing multiple stringent lineage-tracing methods to identify the cell origin for the iNs (Wang et al., 2021b; Rao et al., 2021). Due to the wide variety of cells *in vivo*, compared with the pure culture cells *in vitro*, the origin of the cells *in vivo* should be identified by stringent lineage tracing. Of note, wang et al. found that the AAV-mediated co-expression of NeuroD1 and a reporter efficiently induced reporter-labeled endogenous neurons because NeuroD1 likely cis-regulated the specificity of human GFAP (hGFAP) promoter for its expression in neurons (Wang et al., 2021b). There will be more explorations to figure out complex cross networks among the virus vectors, TFs, and reporters in various cells. They concluded that the tamoxifen-inducible Aldh1l1-CreERT2; R26R-YFP line seems preferable with minimal labeling of endogenous neurons and the Aldh1l1-CreERT2; R26R-tdTomato line also is generally specific by comparing several tracing methods in tracing the lineage of astrocytes (Wang et al., 2021b). At the same time, Rao et al. declared that NeuroD1 and other TFs (PAX6, ASCL1, SOX2, PTBP1) cannot induce microglia-to-neuron conversion via rigid lineage tracing (Rao et al., 2021). It is necessary to employ rigorous lineage tracing in the controversial TFs-mediated cross-lineage reprogramming. Noteworthy, whether there are mislabeling neurons is also needed further investigation during chemical reprogramming. It is misleading to gain exciting results by mislabeling the endogenous neurons (Wang et al., 2021b).

## 6 Administration of Small Molecules *in vivo* Neuronal Reprogramming

Since the microenvironment is complex *in vivo*, it is vital to pursue the optimal delivery strategies of the chemicals. There are several methods for the delivery of the small molecules into the body, such as single intracranial injection, intraperitoneal (i.p.) administration, and constant intracranial injection by an osmotic mini-pumping system, slow-release via biomaterials (Cheng et al., 2015c; Yin et al., 2019; Ma et al., 2021). However, which delivery route is best is a conundrum.

One of the biggest challenges for *in vivo* reprogramming studies is the maintenance of a constant concentration of small molecules inside the brain, and without causing serious damages (Yin et al., 2019). Although core drugs can be sent to the target sites of the brain through stereotaxic intracranial injection, they might cause invasive damage to the brain tissue. In addition, the one-time injection might limit the efficacy of reprogramming due to the inability of maintaining a constant concentration and the limited treatment time. Using intraperitoneal (i.p.) administration, researchers have confirmed that small molecules, such as DAPT, CHIR99021, SB431542, and LDN193189, can pass through the blood-brain barrier (BBB) to regulate adult neurogenesis (Yin et al., 2019). Compared with intracranial injection, intraperitoneal injection is more suitable for repeated administration and has less traumatic effects on rodents. However, repeat high-dose administration of the drug will cause high cost, which might restrain wide application from the clinical translation aspect. Besides, after passing through the BBB, the concentration of small molecules in the intravascular is extremely low, which may affect the *in vivo* efficiency of reprogramming. Therefore, the dosage of small molecules administered by i. p. injection would be much higher than that for intracranial treatment. Furthermore, the *in vivo* dosage of small molecules should usually be higher than that used *in vitro* (Ma et al., 2021), due to part of the drugs permeating the non-target cells *in vivo.* Recently, it has been reported that chemical compounds can be administrated into animal brains (striatum or cortex) at a constant rate *via* a mini-osmotic pumping system that maintains a constant concentration (Gao et al., 2017; Yang et al., 2020c)*.* This method has the advantage of providing a stable and continuous release of the drugs. However, the prolonged presence of mini-osmotic pumps in the brain may cause infection and additional invasive injury to the rodents. In addition, some researchers have proposed to use biomaterial that encapsulates small molecules to achieve a targeted delivery to the brain (Cheng et al., 2015c). Besides, prodrugs could be used to improve the selectivity of the small molecules in the future (Ma et al., 2021). However, the attempt failed since the small molecules did not stay for a long time in the brain, which may be due to the small size and the unsuitability of biomaterial that has been selected for such small molecules.

In summary, no perfect delivery routes have been found for the delivery of small molecules into the brain. An ideal method should be explored in the future to maintain a constant concentration of small molecules in the brain without causing severe invasive damage to the brain, as well as lower off-target effects.

## 7 Direct Neuron Reprogramming in Neurological Disorders

Some studies reported that the conversion of reactive dividing cells, that are found around the lesion sites, into various subtypes of neurons may be a promising strategy for some neurological diseases (An et al., 2018; Mollinari and Merlo, 2021). For example, induced motor neurons can promote recovery of amyotrophic lateral sclerosis (ALS) and spinal cord injury (SCI), and an induced dopaminergic neurons supplement is beneficial to patients with Parkinson’s Disease (PD). Here, we summarize current studies that focused on small molecules-mediated direct neuronal reprogramming in different neurological disease models (Table 3).

**TABLE 3:** Direct neuron reprogramming in neurological disease by small molecules.

Diseases	Starting cells	Desired cells	Media	Small molecules	Signaling pathway	Epigenetic modifications	Metabolic changes	Efficiency	Subtype		Function outcomes	References
AD	Human skin fibroblasts	Human Chemical-induced neurons	DMEM/F12, Neurobasal	VPA, Repsox, CHIR99021, forskolin; SP600125, GO6983, Y27632	mTOR, TGF-β,Wnt, cAMP/PKA,JNK,ROCK	Histone acetylation	Reduce lipid peroxidation, anti-oxidant stress	Human Adult Fibroblasts: 5% (Tuji+)	Glutamatergic		Generate action potentials, form synapses, express glutamate and GABA receptors	Hu et al. (2015)
PD	Adult human retinal pigment epithelial cells	Dopaminergic-like cells	Neurobasal	SB431542, CHIR99021, LDN193189,Y27632	TGF-β, Wnt, BMP/Smad, ROCK, /Smad	—	—	Retinal pigment epithelial cells: 49.87 ± 17.5 ng per 10^6^cells	Dopaminergic		Express FOXA2, express dopamine transporter and	Li et al. (2019)
PD	Human fibroblasts	Dopaminergic neuron-like cells	Neurobasal	VPA, Repsox, kenpaullone, forskolin, purmorphamine Y-27632	mTOR, TGF-β, Wnt, ROCK, cAMP/PKA,SHH	Histone acetylation	Reduce lipid peroxidation, anti-oxidant stress	IMR-90 fibroblasts:: 87.88 ± 2.03% (TUJ1+/DAPI+)	Dopaminergic		Generate action potentials	Qin et al. (2020)
ALS	Spinal cord astrocytes (ALS mouse)	Motor neuron-like cells	Neurobasal, N2, B27	Kenpaullone, forskolin, Y-27632, purmorphamine, RA	Wnt, cAMP/PKA, ROCK,SHH	Histone acetylation	Reduce lipid peroxidation, anti-oxidant stress	Human astrocytes: 86.5 ± 0.5% (TUJ1+HB9+/DAPI+); 83.7 ± 1.9% (TUJ1+ISL1+/DAPI+); ALS mouse astrocytes: 80.0 ± 2.2%	Motor neuron-like cells		Generate action potentials	Zhao et al. (2020)
ALS	Mouse fibroblasts	Motor neurons	Neurobasal, B27	Kenpaullone, forskolin, Y-27632, purmorphamine, RA	Wnt, cAMP/PKA, ROCK,SHH	Histone acetylation	Reduce lipid peroxidation, anti-oxidant stress	Human fibroblasts: 94.56 ± 1.28% (TUJ1+HB9+/DAPI+); 92.46 ± 2.05% (TUJ1+ISL1+/DAPI+),Mouse fibroblasts:>90% (TUJ1+HB9+or TUJ1+ISL1+/DAPI+)	Motor neurons	—		Qin et al. (2018)
SCI	Dermal fibroblasts	Neurons	N3 medium, N2, B27	CHIR99021, Forskolin, LDN193189, SB431542, SP600125, VPA, Y-27632	Wnt, BMP/Smad, TGF-β, cAMP/PKA, ROCK, JNK, mTOR	Histone acetylation	Reduce lipid peroxidation, anti-oxidant stress	Rat dermal fibroblasts: 92.42 ± 0.85% (Tubb3+); Mice dermal fibroblasts:61.34 ± 2.94% ((Tubb3+); Human dermal fibroblasts:47.672. ± 54% (Tubb3+)	—	Generate action potentials		Hu et al. (2019)
SCI	IMR-90 fibroblasts	Motor neurons	Neurobasal, B27	Kenpaullone, Forskolin, Y-27632, Purmorphamine, RA	Wnt, cAMP/PKA, ROCK,SHH	Histone acetylation	Reduce lipid peroxidation, anti-oxidant stress	Human fibroblasts: 94.56 ± 1.28% (TUJ1+HB9+/DAPI+); 92.46 ± 2.05% (TUJ1+ISL1+/DAPI+).Mouse fibroblasts: >90% (TUJ1+HB9+or TUJ1+ISL1+/DAPI+)	Motor neurons	—		Qin et al. (2018)

AD: Alzheimer’s Disease; PD: Parkinson’s Disease; ALS: amyotrophic lateral sclerosis; SCI: spinal cord injury.

Alzheimer’s disease (AD) is a devastating condition of the aging population, characterized by learning and memory deficits due to plaques’ formation in the hippocampus (Kashani et al., 2011). A study has shown that fibroblasts deriving from skins of patients with familial Alzheimer’s disease (FAD) can be directly converted into human chemical-induced neurons (hCiNs) using the chemical cocktail, VCRFSGY, that includes VPA, CHIR99021, Repsox, Forskolin, SP600125, GO6983, Y27632. However, most of the hCiNs are glutamatergic neurons in their induction system (Hu et al., 2015). Regrettably, researchers didn’t conduct chemical transdifferentiation or the transplantation of hCiNs *in vivo* (Hu et al., 2015). What is expected is these hCiNs could be transplanted into the individual patient but don’t cause the risk of tumorigenicity and transplantation rejection, which helps to achieve personalized medicine.

PD is a neurodegenerative disease characterized by the progressive loss of dopaminergic neurons in the midbrain substantia nigra pars compacta (Dawson and Dawson, 2003; Dexter and Jenner, 2013). Motor and nonmotor symptoms are displayed in PD patients, including resting tremors, bradykinesia, postural instability, and rigidity (Hoehn and Yahr, 1967). Li’s team found that adult human retinal pigment epithelial cells can transdifferentiate into dopaminergic-like cells *in vitro* following induction by a combination of SB431542, CHIR99021, and LDN193189. After transplanting the inducted cells into the brain, recipient PD monkeys showed significant improvement in clinical conditions (Li et al., 2019). Another study showed that a cocktail consisting of VPA, Repsox, kenpaullone (another inhibitor of GSK-3β), forskolin, purmorphamine, and Y-27632 can convert human fibroblasts into dopaminergic neuron-like cells *in vitro* (Qin et al., 2020). The transplantation of the induced dopaminergic-like neurons can be utilized to ameliorate Parkinson’s disease in the future. In addition, the direct induction of responsive glial cells in the stratum by small molecules for the generation of dopaminergic-like neurons is another promising research direction.

ALS is a lethal neurodegenerative disorder that affects lower and upper motor neurons (Amico and Antel, 1981). Zhao et al. have found that the drug cocktails KFYPR (kenpaullone, forskolin, Y-27632, purmorphamine, and RA), can induce the transdifferentiation of spinal cord astrocytes, from an ALS mice model carrying a SOD1 mutation, into motor neuron-like cells *in vivo* (Zhao et al., 2020). However, the CiNs exhibited a decrease in cell survival and an increase in oxidative stress compared to wild-type motor neurons derived from healthy mice (Zhao et al., 2020). Another study reported that human fibroblasts can be directly reprogrammed into motor neurons using the same five small molecules *in vitro* (Qin et al., 2018). Moreover, it has been shown that ALS patients derived fibroblasts can be directly converted into motor neurons by introducing the TFs NEUROG2, SOX11, ISL1, and LHX3, and the morphological and survival deficits of ALS human-induced motor neurons (hiMNs) can be ameliorated by the small molecule kenpaullone (Liu et al., 2016).

SCI results in damage to motor, sensory and autonomic functions (Gris et al., 2004; Hou and Rabchevsky, 2014; Smith et al., 2016; Ghasemi-Kasman et al., 2018; Zheng et al., 2021). In 2019, Hu and his colleagues found that after transplantation of neural scaffolds consisting of dermal fibroblasts-reprogrammed neurons that were induced by a small-molecule cocktail CFLSSVY (CHIR99021, Forskolin, LDN193189, SB431542, SP600125, VPA, and Y27632), the damaged tissue was repaired obviously and the hindlimb movements and motor-nerve conductivities of SCI-treated rats were significantly improved (Hu et al., 2019). In addition, human IMR-90 fibroblasts have been directly converted into motor neurons with the small molecules, forskolin, kenpaullone, purmorphamine, RA, and Y-27632 (Qin et al., 2018).

To sum up, most of the current studies focused on the efficiency of different inductive chemical formulas in neuronal reprogramming *in vitro*. Only a few studies observed the *in vivo* transdifferentiation of reactive glial cells into neurons under pathophysiological states. More studies are required to explore the safety and efficacy of endogenous neuronal reprogramming in various neurological disorders from the perspective of future clinical translation.

## 8 Discussion

In the present review, we mainly discussed the mechanisms of different small molecules, the applied approaches in screening small molecule cocktails, the chemical cocktail formulas in neuronal transdifferentiation, the detection methods used to assess the conversion efficiency, and the administration of small molecules *in vivo*. We also summarized current studies that focused on direct small molecules-mediated reprogramming of somatic cells into neurons in different neurological disease models. For the molecular mechanistic studies, we reviewed some pathways involved in chemical reprogramming, such as the Wnt, JAK-STAT, SHH, BMP/Smad, TGF-β, Notch, p38 MAPK, mTOR, cAMP/PKA, JNK, and ROCK pathway. In addition, epigenetic modifications and metabolic changes during the chemical-induced reprogramming process have also been summarized. Some recent scientific tools such as single-cell sequencing and CRISPR-based genome-wide screening will help probe new chemical cocktails and elaborate the underlying induction mechanisms (Wen and Tang, 2016; Joung et al., 2017). At present, the studies about direct neuron reprogramming by small molecules in disease models are rare, with only a few cases in neurodegenerative diseases and SCI. More efforts are needed to explore the application of chemical transdifferentiation in other neurological diseases.

Existing issues and challenges need overcoming in future studies. Firstly, the optimal concentration of each small molecule in the cocktails is difficult to determine. In a study, the researchers tried hundreds of different cocktails tests before finding the optimal concentrations of the nine assessed small molecules (LDN193189, SB431542, TTNPB, thiazovivin, CHIR99021, VPA, DAPT, SAG, and purmorphamine) in converting astrocytes into neurons, which is time-wasting and costly (Zhang et al., 2015). Secondly, the length of the induction time is difficult to determine, as longer times can have toxic effects on the cells, while shorter times might not be effective in inducing neuronal transformation. Thirdly, it is a big challenge to seek an optimal formula to achieve acceptable conversion efficiency. From the clinical translation aspect, the composition of small molecules should be as simple as possible on the premise of acceptable conversion efficiency (Smith et al., 2016; Yin et al., 2019). We summarized the transformation efficiencies from somatic cells to neurons in this review, of which the higher efficiencies were 94.74 ± 0.60% (Tuj+/DAPI+), 94.56 ± 1.28% (Tuj1+HB9+/DAPI+) and 93.7 ± 1.6% (Tuj1+/DAPI+) (Qin et al., 2018; Qin et al., 2020; Zhao et al., 2020). Nevertheless, statistical indicators, cell types and induced protocols of each study were heterogeneous, so it was difficult to clarify the best protocols. More research is needed to explore the most efficient protocols in the future. The *in vitro* microenvironment of induction is relatively simple and easy to monitor. However, there are many unresolved questions vis-à-vis the *in vivo* application of small molecules-mediated programming. Specifically speaking, how the *in vivo* complex microenvironment interacts with small molecules and whether it changes the effect of small molecules, are questions that require further investigations. Moreover, since small molecules lack cellular specificity to enter the brain, it is hard to judge whether other cell types, apart from the intended starting cell type (Man et al., 2018), are affected. In addition, some of the chemicals may have some unwanted toxic effects on normal brain cells (Man et al., 2018). Another major concern is whether newly transformed neurons can survive for a long time and efficiently integrate into local neural circuits. Some researchers have adopted retrograde a mono-transsynaptic tracing technique using a pseudotyped rabies virus to determine whether CiNs can form synaptic connections with host neurons *in vivo* (Ma et al., 2021). Another challenge for *in vivo* chemical transdifferentiation is the development of an ideal administration method that preserves a constant concentration of small molecule compounds inside the brain without causing severe invasive damage. A micro-injection pump for continuous drug delivery and biological material for drugs’ slow-release are attractive tools for the delivery of the drugs (Yin et al., 2019; Ma et al., 2021). To achieve the cellular specificity of small molecules, nanoparticles containing specific signals which can recognize target cells can be utilized in the future (Prapainop et al., 2012). For the treatment of some neurological disorders, subtype-specific neurons should be induced in appropriate regions of the brain (Smith et al., 2016). Recently, it has been proposed that some *in vitro* chemical-induced stable neurons could be constructed for brains by a three-dimensional (3D) bioprinting technology, which prints biodegradable materials with cells as 3D tissue (Ho and Hsu, 2018; Yuan et al., 2020). In 2021, a lab bioprinted MSC-derived neural tissues successfully using a fibrin-based bioink and the RX1 bioprinter with the aid of SB431542, LDN-193189, purmorphamine, fibroblast growth factor 8 (FGF8), fibroblast growth factor-basic (bFGF), and brain-derived neurotrophic factor (BDNF) (Restan Perez et al., 2021). Even more interesting is that Ho et al. achieved cell reprogramming via 3D bioprinting of human fibroblasts in polyurethane hydrogel for the manufacture of neural-like constructs with Forkhead box D3 (FoxD3), a transcription factor (Ho and Hsu, 2018).

Since *in vivo* reprogramming of glial cells into neurons is an excellent research avenue for the treatment of neurological disorders, a better understanding of the mechanisms of small molecule cocktails-induced neuronal reprogramming is critical. However, there are currently limited studies that were published on the endogenous transdifferentiation from somatic cells into neurons and in different disease models. Future research efforts are needed to screen the optimal induction formula, attain cellular specificity entry of small molecules, optimize the routes of administration and improve transdifferentiation efficiency.

## Nomenclature

### Abbreviations

AAV-FLEX-EGFP: flexed Enhanced Green Fluorescent Protein-adeno-associated virus
AD: Alzheimer’s Disease
ALDH1L1: Aldehyde dehydrogenase 1 family member L1
ALK: Activin-receptor-like kinase
ALS: amyotrophic lateral sclerosis
ATRA: all-trans retinoic acid
BBB: blood-brain barrier
BDNF: brain-derived neurotrophic factor
bFGF: fibroblast growth factor-basic
BMP: bone morphogenetic protein
cAMP: cyclic adenosine monophosphate
CAYTFVB: CHIR99021, A8301, Y27632, TTNPB, Forskolin, Valproic acid (VPA), NaB
CFLSSVY: CHIR99021, Forskolin, LDN193189, SB431542, SP600125, VPA, and Y27632
CiNs: Chemically-induced neurons
CNS: Central Nervous System
CREB: cyclic AMP-responsive element-binding protein
CRISPR/Cas9: Clustered regularly interspaced short palindromic repeat/Cas9
CRISPR: Clustered Regularly Interspaced Short Palindromic Repeats
DCX: doublecortin
DEGs: differentially expressed genes
DFICBY: DBcAMP, Forskolin, ISX9, CHIR99021, I-BET151, Y-27632
DNA: deoxyribonucleic acid
DNMTs: DNA methyltransferases
EMT: Epithelial-to-Mesenchymal transition
ERK: extracellular signal-regulated kinases
FAD: familial Alzheimer’s disease
FGF8: fibroblast growth factor 8
FICSB: Forskolin, ISX9, CHIR99021, SB431542, and I-BET151
FoxD3: Forkhead box D3
FRSCGYIB: Forskolin, RepSox, SP600125, CHIR99021, Go6983, Y27632, IXS9, I BET151
GAPs: GTPase-activating proteins
GEFs: guanine nucleotide-exchange factors
GFAP: glial fibrillary acidic protein
GFP: green fluorescent protein
GO: Gene Ontology
GSK3: glycogen synthase kinase-3
H3K27: histone H3 at lysine 27
H3K9: histone H3 at lysine 9
HAT: histone acetyltransferases
hCiNs: human chemical-induced neurons
HDACs: histone deacetylases
hGFAP: human GFAP
HIF-1α: Hypoxia-inducible factor 1-alpha
hiMNs: human-induced motor neurons
iNs: Induced neurons
iNSCs: Induced multipotent neural stem cells
iPSCs: Induced pluripotent stem cells
JAG1/2: jagged 1/2
JAK-STAT: Janus family tyrosine kinase–signal transducer and activator of transcription
JNK: Jun N-terminal Kinase
KFYPR: kenpaullone, forskolin, Y-27632, purmorphamine, RA
LRP5/6: receptor-related protein 5/6
MAP2: microtubule association protein-2
MAPKs: mitogen-activated protein kinase
MET: Mesenchymal-to-Epithelial transition
MSCs: mesenchymal stromal cells
mTOR: Mammalian Target of Rapamycin
NeuN: neuronal nuclei
NF-κB: nuclear factor kappa B
NICD: Notch intracellular domain
NPCs: neural progenitor cells
OXPHOS: oxidative phosphorylation
PD: Parkinson’s Disease
PTCH1: patched 1
RA: retinoic acid
RBPJ: recombining binding protein suppressor of hairless
RNA-seq: RNA-sequencing
ROCK: Rho-associated protein kinase
ROS: reactive oxygen species
RT-qPCR: real-time quantitative polymerase chain reaction
SCI: spinal cord injury
SH2: Src homology 2
SHH: Sonic Hedgehog
SLCD: SB431542, LDN193189, CHIR99021, DAPT
SMO: smoothened
sPSCs: spontaneous postsynaptic currents
TCF/LEF1: T-cell factor/lymphoid enhancer factor
TFs: Transcription factors
TGF-β: transforming growth factor-beta
TSS: transcription start site
TUJ1: β-tubulin III
VCRF: VPA, CHIR99021, Repsox, Forskolin
VCRFSGY: VPA, CHIR99021, Repsox, Forskolin, SP600125, GO6983, Y27632
VCRFSGYIBRTQ: VPA, CHIR99021, Repsox, Forskolin, SP600625, GO6983, Y27632, ISX9, I-BET151, RA, Vit C, QVD-OPH
Wnt: Wingless/integrated
